# The submandibular and sublingual glands maintain oral microbial homeostasis through multiple antimicrobial proteins

**DOI:** 10.3389/fcimb.2022.1057327

**Published:** 2023-01-10

**Authors:** Yanan Li, Jingming Liu, Tong Guan, Yuxin Zhang, Qianyu Cheng, Huikai Liu, Chang Liu, Wenping Luo, Hong Chen, Liang Chen, Tianyu Zhao

**Affiliations:** ^1^ Chongqing Key Laboratory of Oral Diseases and Biomedical Sciences, Stomatological Hospital of Chongqing Medical University, Chongqing, China; ^2^ Department of Endodontics, Stomatological Hospital of Chongqing Medical University, Chongqing, China; ^3^ Stomatological Hospital of Chongqing Medical University, Chongqing, China; ^4^ Chongqing Municipal Key Laboratory of Oral Biomedical Engineering of Higher Education, College of Stomatology, Chongqing Medical University, Chongqing, China; ^5^ First Clinical College, Chongqing Medical University, Chongqing, China

**Keywords:** submandibular glands, sublingual glands, saliva, oral microbial homeostasis, antimicrobial proteins

## Abstract

**Introduction:**

Oral microbial homeostasis is a key factor affecting oral health, and saliva plays a significant role in maintaining oral microbial homeostasis. The submandibular gland (SMG) and sublingual gland (SLG) together produce the most saliva at rest. Organic ingredients, including antimicrobial proteins, are rich and distinctive and depend on the type of acinar cells in the SMG and SLG. However, the functions of the SMG and SLG in maintaining oral microbial homeostasis have been difficult to identify and distinguish, given their unique anatomical structures

**Methods:**

In this study, we independently removed either the SMG or SLG from mouse models. SMGs were aseptically removed in three mice in the SMG-removal group, and SLGs were aseptically removed in three mice in the SLG-removal group. Three mice from the sham-operated group were only anesthetized and incised the skin. After one month, we analyzed their oral microbiome through 16S rRNA sequencing. And then, we analyzed each gland using proteomics and single-cell RNA sequencing.

**Results:**

Our study revealed that the microbiome balance was significantly disturbed, with decreased bacterial richness, diversity, and uniformity in the groups with the SMG or SLG removed compared with the sham-operated group. We identified eight secreted proteins in the SMG and two in the SLG that could be involved in maintaining oral microbial homeostasis. Finally, we identified multiple types of cells in the SMG and SLG (including serous acinar, mucinous acinar, ductal epithelial, mesenchymal, and immune cells) that express potential microbiota homeostasis regulatory proteins. Our results suggest that both the SMG and SLG play crucial roles in maintaining oral microbial homeostasis via excretion. Furthermore, the contribution of the SMG in maintaining oral microbial homeostasis appears to be superior to that of the SLG. These findings also revealed the possible antimicrobial function of gland secreta.

**Discussion:**

Our results suggest that control of oral microbial dysbiosis is necessary when the secretory function of the SMG or SLG is impaired. Our study could be the basis for further research on the prevention of oral diseases caused by microbial dysbiosis.

## Introduction

A balanced oral microbiome is important for normal functioning of the body (Baker et al., 2014). Distorted microbial homeostasis leads to the prevalence of pathogenic bacteria, which cause various diseases (Lynge Pedersen and Belstrom, 2019). Previous studies have connected oral microbiome dysbiosis and oral diseases, including dental caries, gingivitis, and periodontitis (Cross and Ruhl, 2018). Recently, studies have demonstrated links to systemic diseases, including autoimmune diseases, systemic malignancies, and premature birth (Alam et al., 2020; Freire et al., 2021).

Several factors influencing oral microbial homeostasis are well understood, including host factors, local environment, and factors associated with the microorganisms themselves (Lynge Pedersen and Belstrom, 2019). Saliva plays a significant role in the maintenance of oral microbial homeostasis, having dual effects on oral microbiome growth (Carpenter, 2020). Some salivary proteins, such as lysozymes, lactoferrins, peroxidases, mucins, immunoglobulins, histatins, cystatins, and amylases, can inhibit growth. Immunoglobulins participate in locally acquired immunity, whereas lysozymes, peroxidases, and mucins participate in local innate immunity (Saitou et al., 2020). Conversely, other salivary proteins, such as mucins, can aggregate bacteria, causing them to adhere to solid surfaces, thus acting as a medium for oral microbial growth (Lynge Pedersen and Belstrom, 2019).

In mammals, saliva is predominantly synthesized and secreted by three major pairs of anatomically and histologically distinct organs: the parotid gland (PG), submandibular gland (SMG), and sublingual gland (SLG) (Oyelakin et al., 2019). The composition and function of secreta from each gland is unique, consisting of different cell types (Mattingly et al., 2015). Most saliva produced in response to stimuli is secreted by the PG, whereas in the resting state saliva is produced by the SMG and SLG, which play key roles in maintaining oral microbial balance. PG (a purely serous gland) produces watery, α-amylase-rich saliva, whereas the SMG (which contains mucous and serous acinar cells) and SLG (which contains mainly mucous acinar cells) produce more viscous, slimy, mucin-rich secretions (Mattingly et al., 2015). Saliva constituents in the SMG and SLG are similar but not identical; Mucin levels in the SLG are 10-fold higher than in the SMG. Additionally, these mucins have different chemical properties. Nearly all sialic acids are mucin-bound in the SLG, whereas only 40% are mucin-bound in the SMG (Porcheri and Mitsiadis, 2019).These differences suggest distinct functions in maintaining oral health.

Functionally, proteins are the paramount constituents of saliva (Cross and Ruhl, 2018; Saitou et al., 2020). Previous studies focused on acinar cells, the predominant source of salivary proteins (Saitou et al., 2020). The heterogeneity of protein-secreting cells in the SMG and SLG has recently been discovered using single-cell sequencing technology (Hauser et al., 2020). However, the proteins that regulate microbiota homeostasis and the cells where they are synthesized remain undefined. Human ductal anatomy prevents proteomic analysis of pure submandibular or sublingual secretions. Murine models are used due to similarities in saliva constituents, but the amount of secreta collected from mice is insufficient for proteomic analysis. Therefore, the mechanisms by which the SMG and SLG directly or indirectly affect microbiota homeostasis remain unclear.

To investigate this, we removed SMG or SLG from mouse models and employed 16S rRNA sequencing to detect changes in oral microflora. We also performed proteomic analyses to identify antimicrobial proteins. Additionally, single-cell RNA sequencing (scRNA-seq) was conducted to explore the cell types responsible for secreting antimicrobial proteins.

Our results deepen our understanding of the relationship between antimicrobial proteins in the SMG/SLG and oral microbial homeostasis, and highlight differences between the SMG and SLG in this role. Our results suggest that control of oral microbial dysbiosis is crucial when SMG or SLG secretory function is impaired. This study offers prospects for preventing diseases caused by microbial dysbiosis *via* the application of antimicrobial proteins into the oral cavity.

## Materials and methods

### Animals’ treatment

Female C57BL/6N mice (6–8 weeks old) were purchased from Beijing Vital River Laboratory Animal Technology Co. Ltd. (Certificate No. 110011210109849878) and raised in a Specific pathogen Free grade environment with a constant photoperiod (12 h light/12 h darkness). All animal studies were approved by the Ethics Committee of Chongqing Medical University College and Use Committee (grant number: 2021062). The Guidelines for Ethical Review of Laboratory Animal Welfare (GB/T35892-2018, China) were followed at our institution. In this study, all experiments were performed in accordance with the Basel Declaration and followed the “3R” principles of treating experimental animals: reduction, replacement, and refinement.

### Establishment of submandibular glands or sublingual glands removal model and saliva collection

**Figure 1:**
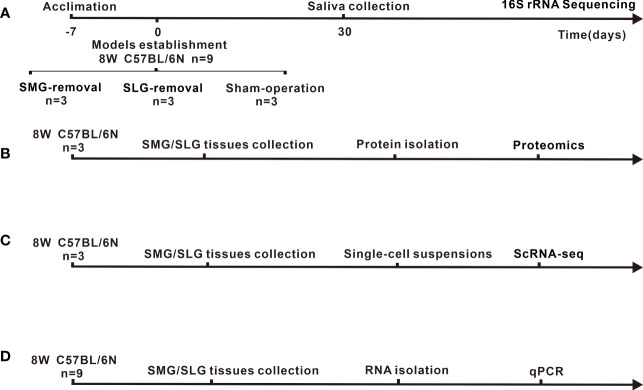
Experimental timeline and animal treatment flowchart. **(A)** Sample source, model-establishing period and sampling time point of 16S rRNA for sequencing. **(B)** Source of samples and protocol flowchart for proteomics to investigate secreted antimicrobial proteins of SMG or SLG. **(C)** Source of samples and animal treatment flowchart for ScRNA-seq to explore heterogenous cells of SMG or SLG secreted antimicrobial proteins are presented. **(D)** Source of samples and flowchart for qPCR to examine the transcription of secreted antimicrobial proteins.

Mice were anesthetized with an intraperitoneal injection of 400 mg/kg of 5% chloral hydrate. After disinfection with 75% alcohol, the submandibular area was exposed. SMGs were aseptically removed in three mice in the SMG-removal group, and SLGs were aseptically removed in three mice in the SLG-removal group. Three mice from the sham-operated group were only anesthetized and incised the skin (Hori et al., 2021). After one month, the oral bacteria was collected from the mouse oral cavity by swabbing the surface of the oral mucosa and collecting saliva with sterilized cotton balls. Saliva flow was stimulated (subcutaneous 5 mg/kg pilocarpine), and saliva was collected using sterile cotton balls. Samples were obtained after centrifugation (12000 rpm, 5 min) and stored at −80 °C for further analysis. The timeline of the experiment as shown in **Figure 1A**. The exclusion criteria were as follows: (a) loss of ability to ambulate (inability to access food or water). (b) animals that died during the study period (Hori et al., 2021).

### DNA preparation and 16S rRNA sequencing

Total DNA was extracted using the Omega Mag-Bind Soil DNA Kit (Omega, Georgia, USA) according to the manufacturer’s protocol. DNA concentration was assessed using Nanodrop 2000 (Thermo Scientific, Wilmington, USA), and the DNA quality was determined by 1.2% agarose gel electrophoresis. The 16S rRNA sequencing was performed by Genergy Biotechnology (Shanghai, China). Sequences that reflect the composition and diversity of bacterial flora, such as microbial ribosomal RNA or specific gene fragments, were used as targets, and sample-specific barcode sequences were added to design the corresponding primers. The V3-V4 hypervariable regions of the bacterial 16S rRNA gene were amplified by PCR using Pfu high-fidelity DNA polymerase (Quanshijin Company, Beijing, China). The products of PCR amplification were quantified by fluorescence (Quant-iT PicoGreen dsDNA Assay Kit) (Invitrogen, Shanghai, China) using a Microplate reader (BioTek, FLx800). Sequencing libraries were prepared using a TruSeq Nano DNA LT Library Prep Kit (Illumin, California, USA). The final fragments were selected and purified by 2% agarose gel electrophoresis.

### Bioinformatics analysis of 16S rRNA sequencing

Microbiome bioinformatics were mainly performed using QIIME 2 2019.4 (Bokulich et al., 2018), while the OTU clustering procedure followed Vsearch (v2.13.4) (Rognes et al., 2016). All unique sequences were clustered at 98% (via cluster_size), followed by chimera removal (via uchime_denovo). Non-chimeric sequences were re-clustered at 97% to generate representative OTU sequences and OTU tables. The representative sequences were aligned with MAFFT (Katoh et al., 2002) and used to construct a phylogeny using fasttree2 (Price et al., 2009). Alpha diversity analysis was performed by calculating indices of Chao1, Simpson, and Pielou’s evenness, beta diversity metrics (weighted UniFrac) were estimated using the diversity plugin with samples (Lozupone et al., 2007). Rarefaction curves and the total OTUs are shown in **Appendix Figure 1**. We removed any OTUs that are less than 1% of the total abundance and unidentified genera when we did differential abundance analysis.

### Sample collection and bioinformatics analysis of proteomics

The source of samples as shown in **Figure 1B**. A total of six submandibular and sublingual glands were collected for proteomic analysis, obtained from three C57BL/6N mice, which were euthanized using CO2. The submandibular and sublingual glands were washed with sterile phosphate-buffered saline to rinse the blood and adhesive tissue. Samples were sent to Genecreate Biological Engineering Co., Ltd (Wuhan, China) for proteomic analysis. Genecreate Biological Engineering Co., Ltd uses label-free quantification to detect the differences within the same protein among multiple samples, including protein extraction, reductive alkylation, protein quantification, enzyme de-salting, Q-Exactive HF (Thermo Fisher Scientific, San Jose, CA) mass spectrometry system analysis, database retrieval analysis using MaxQuant 1.6.17.0 software. In the quantitative results, the significant difference was selected according to up_regulate ≥2 or down_regulate ≤0.500, adj. p-value ≤0.05. Principal coordinate analysis (PCoA) was performed using Bray-Curtis distance metrics. Functional enrichment analysis included biological process category of the gene ontology (GO), Kyoto Encyclopedia of Genes and Genomes (KEGG) pathway, and InterPro (IPR). They were performed for all proteins with significant differences. The database of KEGG pathways from http://www.kegg.jp /. The database of GO from http://www.geneontology.org /.

### Quantitative real-time polymerase chain reaction (qPCR)

The source of samples are shown in **Figure 1D**. RNA from the SMG and SLG of nine C57BL/6N mice (6–8 weeks old, female) were isolated using TRIZOL reagent (15596026, Invitrogen, Carlsbad, CA, USA) and used to generate cDNA templates using a reverse transcription reaction kit (18090050, Invitrogen, Carlsbad, CA, USA). The PCR primers were designed using Primer 3.0 and are listed in **Table 1 of Appendix**. SYBR Green-based qPCR analysis was conducted using the ABI Prism 7500 Real-Time PCR System (Applied Biosystems, Foster City, CA, USA). PCRs were tested using 20-µl reactions. qPCR cycles were 95°C for 3 min, followed by 39 cycles at 95°C for 10 s, 59°C for 30 s, and one cycle at 95°C for 5 s, 65°C to 95°C, incremented by 0.5°C for 5 s. qPCR reactions were performed in triplicate. All samples were normalized to the β-actin expression levels. Sample quantification was performed according to the threshold cycle method using the ΔΔCt method. Values presented in the graphs are mean ± SEM values.

### Single-cell RNA sequencing (scRNA-seq) analysis

The source of scRNA-seq samples are shown in **Figure 1C**. Single-cell suspensions from freshly isolated SMGs and SLGs from three female C57BL/6N (6–8 weeks old) mice were generated for scRNA-seq analysis as previously described (Song et al., 2018). A total of 15,707 cells were sequenced at a depth of 295 million reads, with a mean of 18,816 reads per cell and 689 median genes per cell. The output from the 10X Genomics Cell Ranger v6.0.1 pipeline was used as input into the R analysis package Seurat v4.1.1 (Hao et al., 2021). Data were normalized using Seurat’s LogNormalize with a scale factor of 10,000. Cluster analysis was performed, and the t-distributed stochastic neighbor embedding algorithm was used for dimensionality reduction and visualization. Cluster-to-cluster differential expression testing using the Wilcoxon rank-sum test identified unique gene markers for each cluster.

## Results

### Richness, diversity, and uniformity of the oral microbiome decreased in the SMG- or SLG-removal model

To investigate the role of the SMG and SLG in maintaining oral microbiome balance, we established SMG- or SLG-removal mouse models and analyzed their microbial composition. The results of the blood test (**Appendix, Table 2**) showed that the monocyte ratio decreased in the SMG-removal group, and the basophil ratio increased in the SLG-removal group. We performed high-throughput sequencing of the 16S rRNA gene V3-V4 regions of saliva samples. Sequencing yielded approximately 109,115 valid reads for each sample, after removing low-quality or ambiguous reads. Interestingly, we found significant differences in oral microbial composition.

**Figure 2:**
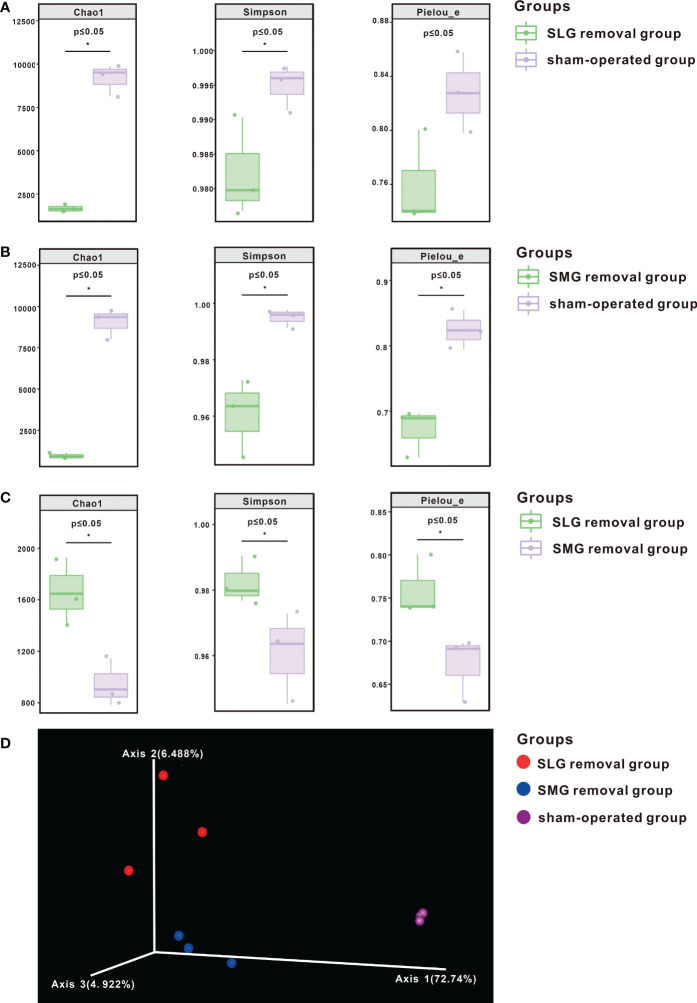
The diversity of bacterial species shown by alpha diversity indices based on 16S rRNA sequencing. The Chao1 index represented richness, the Simpson index represented diversity, and Pielou’s Evenness index characterized uniformity. **(A)** These indices were significantly decreased (P ≤ 0.05) in the SLG removal group compared with the sham-operated group. **(B)** These indices were significantly decreased (P ≤ 0.05) in the SMG removal group compared with the sham-operated group. **(C)** These indices were significantly decreased (P ≤ 0.05) in the SMG removal group compared with the SLG removal group. **(D)** Beta diversity is shown by PCoA. Each sample is represented by a dot, and different colors represent different groups. A separation trend was shown among the three groups. The symbol * means P≤0.05.

To comprehensively evaluate the alpha diversity of the microbial community, significance among the three groups was verified using the Kruskal-Wallis rank sum test and Dunn’s test. As shown in **Figures 2A, B** the indices were significantly lower (P ≤ 0.05) in both the SMG-removal and the SLG-removal groups than in the sham-operated group. Alpha diversity indices were also significantly lower in the SMG-removal group than in the SLG-removal group (P ≤ 0.05) (**Figure 2C**). These results suggest that regulation of the oral microbiome is complex and may differ between the two glands.

Beta diversity was measured using a weighted UniFrac distance matrix and analyzed using PCoA. The visualization results are shown in **Figure 2D**. A separation trend was observed among SMG-removal group, SLG-removal group, and sham-operated group suggesting that the composition of the oral microbiome was distinctive among the three groups of mice.

### *Lactobacillus* was the most clearly increased genus in the SMG- and SLG-removal models

**Figure 3:**
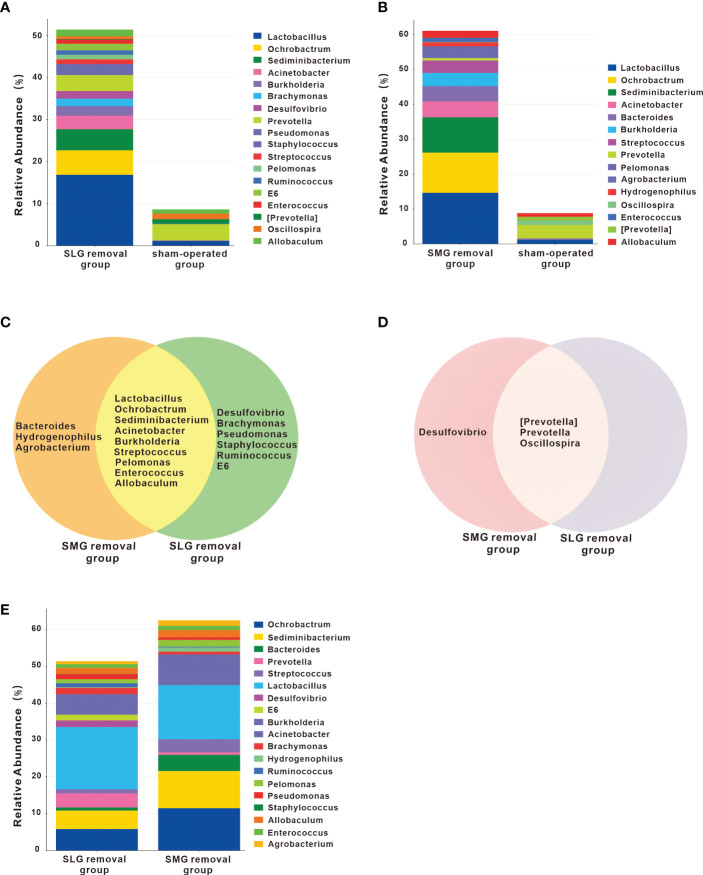
Changes of genera in the SMG or SLG removal group. **(A)** The different genera compared the SLG removal group with the sham-operated group. The most obviously up-regulated genus was Lactobacillus. **(B)** The different genera were found when comparing the SMG removal group with the sham-operated group. The most obviously up-regulated genus was also Lactobacillus. **(C)** Up-regulated genera either in the SMG or in the SLG removal group, compared with the sham-operated group. Co-upregulated genera were Lactobacillus, Ochrobactrum, Sediminibacterium, Acinetobacter, Burkholderia, Streptococcus, Pelomonas, Enterococcus, and Allobaculum. Bacteroides, Hydrogenophilus and Agrobacterium only occurred in the SMG removal group, while Desulfovibrio, Brachymonas, Pseudomonas, Staphylococcus, Ruminococcus, E6 only occurred in the SLG removal group. **(D)** Down-regulated genera either in the SMG or in the SLG removal group, compared with the sham-operated group. Co-downregulated genera were [Prevotella], Prevotella and Oscillospira. Desulfovibrio existed only in the SMG removal group. **(E)** The different genera compared the SLG removal group with the SMG removal group. The abundance of Lactobacillus was more prominent in the SLG removal group compared with the SMG removal group. The abundance of streptococcus was more in the SMG removal group compared with the SLG removal group.

We compared the genera among the three groups by differential abundance analysis (**Figures 3A, B, E**). The genera that differed between the SLG-removal group and the sham-operated group are shown in **Figure 3A**. The genera that differed between the SMG-removal group and the sham-operated group are shown in **Figure 3B**. Moreover, the genera that differed between the SLG-removal group and SMG-removal group are shown in **Figure 3E**. From these results, we concluded that *Lactobacillus* was the most variable genus in the gland-removal groups. Both *Lactobacillus* and *Desulfovibrio* were upregulated, whereas *Streptococcus* was downregulated in the SLG-removal group compared to the SMG-removal group. Next, we evaluated the up- or downregulated genera from this genera in both the SMG and SLG removal groups compared with the sham-operated group (**Figures 3C, D**). We identified nine genera of co-upregulated microflora and three genera of co-downregulated microflora. *Desulfovibrio* was only upregulated in the SLG-removal group and was downregulated in the SMG-removal group.

### Several proteins secreted in the SMG and SLG may participate in maintaining oral microbial homeostasis

**Figure 4:**
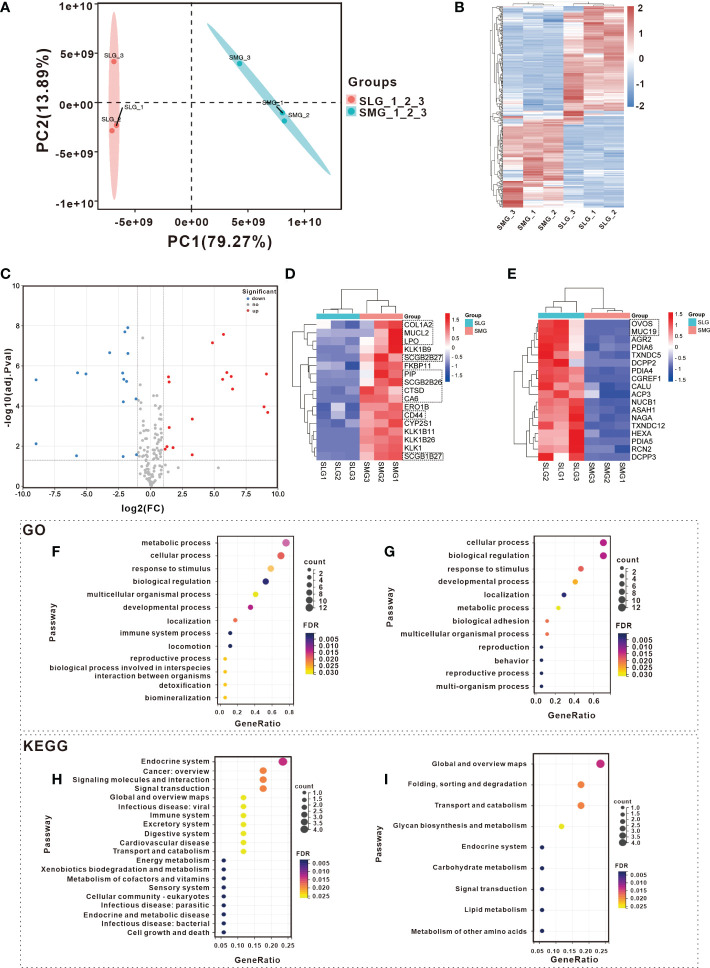
Detection and analysis of proteins in SMG and SLG which participate in maintaining oral microbial homeostasis with Proteomic. Different expression tissue proteins between SMG and SLG were shown with **(A)** PCoA and a **(B)** heatmap. A separation trend was displayed between SMG and SLG. There were 114 up-regulated and 134 down-regulated proteins in the SMG compared to the SLG. **(C)** The secreted proteins of the SMG and the SLG were displayed with a volcano plot. There were 17 up-regulated and 18 down-regulated proteins in the SMG compared with the SLG. Secreted proteins were more abundant in the **(D)** SMG and more abundant in the **(E)** SLG. The biological process of GO analysis was conducted in more abundant expression proteins of **(F)** SMG and **(G)** SLG. The KEGG annotation analysis was conducted in more of the abundant expression proteins of the **(H)** SMG and **(I)** SLG. The GO and KEGG pathway enrichment of the up-regulated proteins, which were more abundantly expressed in the SMG were likely enriched secondary to infectious disease or immunity. While the down-regulated proteins, which were more abundantly expressed in the SLG in GO and KEGG enrichment, did not show that.

Proteomic assays were performed to examine the protein composition of the two glands. A total of 3,718 proteins were detected based on 36,331 peptides. The differences between the SMG and SLG are displayed using PCoA and a heatmap. The PCoA plot of the SMG and SLG showed an obvious separation, with the most decisive PC1 accounting for 79.27% of the variation in the dataset (**Figure 4A**). This suggests that the protein composition differs between the SMG and SLG. A total of 248 significant hits (114 upregulated and 134 downregulated) were found in the SMG compared to the SLG (upregulated ≥ 2 or downregulated ≤ 0.500, adj. P-value ≤ 0.05) (**Figure 4B**).

We then focused on the secreted proteins. A total of 309 proteins, characterized by the enrichment of extracellular proteins and signal peptides, were selected for further analysis. Thirty-five proteins exhibited significant differences (17 upregulated and 18 downregulated) (**Figure 4C**). The heatmap displays relatively abundant proteins in the SMG (**Figure 4D**) and SLG (**Figure 4E**). The biological process category of the GO and KEGG pathway enrichment analyses were determined to explore the function of the 35 significantly altered proteins (**Figures 4F–I**). The GO terms of the upregulated proteins, which were more abundantly expressed in the SMG, were enriched in the immune system process category (**Figure 4F**). KEGG pathway enrichment revealed that 17 upregulated proteins that were more abundantly expressed in the SMG were enriched with “infectious disease: viral,” “immune system,” “infectious disease: parasitic,” and “infectious disease: bacterial” (**Figure 4H**). Meanwhile, the proteins that were more abundant in the SLG were not relevant to infectious diseases or immunity according to the GO and KEGG enrichment analyses (**Figures 4G, I**). These results might partially explain why bacterial richness, diversity, and uniformity were more disrupted in the SMG-removal group than in the SLG-removal group.

Next, we analyzed the detailed functions of the selected proteins by enriching IPR, KEGG, and GO functions. Seventeen proteins were more abundant in the SMG. Prolactin-inducible protein (PIP) has an immunoglobulin fold-like structure that contributes to the aggregation of oral bacteria. Aggregation is thought to promote the clearance of bacteria from the oral cavity and can influence the composition of the oral bacterial community. Lactoperoxidase (LPO), a member of the salivary peroxidase system, exerts a broad-spectrum bactericidal effect (Morita et al., 2017). CTSD (cathepsin D), CD44 (CD44 antigen), and COL1A2 (collagen alpha-2) were associated with the pathways of “infectious disease: viral” and “immune system”. Additionally, SCGB2B26, SCGB1B27, and SCGB2B27 were subjected to IPR enrichment and were found to belong to the Secretoglobin superfamily. Eighteen proteins were more abundant in the SLG. As salivary mucin, MUC19 lubricates and protects oral surfaces and assists in bacterial clearance by promoting the aggregation of microorganisms by interacting with other salivary proteins. Ovostatin homolog (OVOS) was subjected to IPR enrichment and was found to belong to the immunoglobulin E set. Interestingly, a non-secreted protein, the polymeric immunoglobulin receptor (PIGR), has been reported to be positively correlated with IgA concentrations in mouse saliva (Huang et al., 2018; Gong et al., 2021).

**Figure 5:**
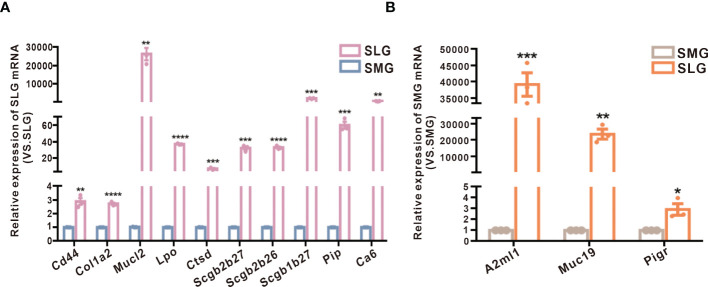
Examination of protein transcription in the SMG and the SLG by qPCR. **(A)** The transcript levels of *Lpo, Ctsd, Cd44, Col1a2, Pip, Scgb2b26, Scgb1b27*, and *Scgb2b27* in the SMG and the SLG tested by qPCR. **(B)** The transcript levels of *Muc19, A2ml1* (coding protein OVOS), and *Pigr* in the SMG and the SLG were tested by qPCR. The transcript levels were consistent with proteins abundance. The meaning of the symbols: *p≤0.05, **p≤0.01, ***p≤0.001, ****p≤0.0001.

To further confirm these results, we conducted a qPCR to examine protein transcription in the SMG and SLG (**Figures 5A,B**). The results showed that the SMG’s expression of *Lpo*, *Ctsd*, *Cd44*, *Col1a2*, *Pip*, *Scgb2b26*, *Scgb1b27*, and *Scgb2b27* was higher, whereas the expression of *Muc19*, *A2ml1* (codes for the OVOS protein), and *Pigr* was higher in the SLG. These RNA transcripts were consistent with the proteomic results.

### Heterogenous cells of the SMG or SLG secreted antimicrobial proteins

**Figure 6:**
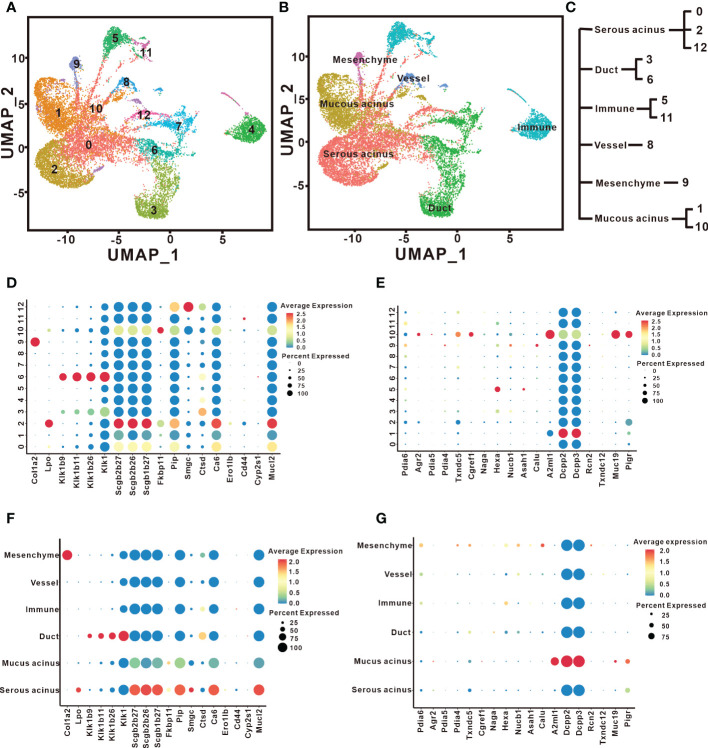
Cell types responsible for distinct secreted proteins identified by scRNAseq. **(A)** UMAP visualization revealed twelve cell clusters in the SMG and the SLG. **(B)** The distribution and cell types in the SMG and the SLG include serous acinar cells, mucinous acinar cells, ductal epithelial cells, immune cells, vascular cells, and mesenchymal cells. **(C)** The relationship between the 12 clusters and these six cell types. **(D, F)** The expression patterns of genes coding the secreted proteins that are more abundant in SMG are shown by a bubble plot. **(E, G)** The expression patterns of genes coding the secreted proteins that are more abundant in the SLG are shown by a bubble plot.

To explore the types of cells that generate different proteins in the SMG and SLG, we conducted scRNA-seq assays. First, we performed unsupervised clustering with affinity propagation based on the expression of high-variance genes to determine the level of cellular heterogeneity in the SMG and SLG. Twelve clusters were identified using the uniform manifold approximation and projection (UMAP) (**Figure 6A**). Six cell clusters were assigned according to classical cellular markers (**Figure 6B**): serous acinar cells marked by lactoperoxidase (Huang et al., 2021), mucinous acinar cells marked by mucin19 (Das et al., 2009), ductal epithelial cells marked by keratin 7 (Rimland et al., 2021), immune cells marked by natural killer cell granule protein 7 (Ng et al., 2020), vascular cells marked by cadherin 5 (Tombor et al., 2021) and mesenchymal cells marked by collagen type I alpha 1 (Tombor et al., 2021). The relationship between the 12 clusters and the six cell types is shown in **Figure 6C**. Cluster 0, cluster 2, and cluster 12 were assigned to serous acinar cells. Cluster 1 and cluster 10 were assigned to mucinous acinar cells. Cluster 3 and cluster 6 were assigned to the ductal epithelial cells. Cluster 5 and cluster 11 were assigned to immune cells. Cluster 8 was assigned to vascular cells. Cluster 9 was assigned to mesenchymal cells.

Next, we generated an expression map of the proteins secreted by the six cell types. Antibacterial proteins abundantly expressed in the SMG were carefully analyzed (**Figures 6D, F**). The results showed that *Lpo*, *Scgb2b27*, *Scgb2b26*, *Scgb1b27*, *Mucl2*, and *Pip* were highly expressed in serous acinar cells. *Ctsd* is highly expressed in ductal epithelial cells. Thus, the secretory units express most antibacterial proteins. We also found that some secreted antibacterial proteins were present in multiple cell types. For instance, *Col1a2* is highly expressed in mesenchymal cells, and *Cd44* is highly expressed in immune cells. Two SLG candidates were analyzed. *A2ml1* (codes for the OVOS protein) and *Muc19* were highly expressed in mucinous acinar cells (**Figures 6E, G**). Interestingly, *Pigr* is also highly expressed in mucinous acinar cells. Thus, more cells and proteins appeared to be involved in regulating bacterial richness, diversity, and uniformity in the SMG than in the SLG. Overall, our results illustrate those serous acinar, ductal epithelial, mesenchymal, and immune cells in the SMG produce antimicrobial proteins, whereas only mucinous acinar cells in the SLG produce antimicrobial proteins.

## Discussion

Many antimicrobial proteins required for maintaining oral microbial homeostasis have been identified. However, details regarding the cellular sources and functions of these proteins are poorly understood. We performed 16S rRNA sequencing of the salivary microbiome, and employed proteomics and scRNA-seq to study the functional proteins and antimicrobial protein-producing cell populations. We found that these proteins may regulate bacterial richness, diversity, and uniformity. Proteomic analysis showed that eight proteins secreted in the SMG and two proteins secreted in the SLG may be related to oral microbial homeostasis. scRNA-seq revealed that these antimicrobial proteins originate from serous acinar, ductal epithelial, mesenchymal, immune, or mucinous acinar cells.

Regulation of the oral microbiome differs between the SMG and SLG. Removing SMG or SLG (especially SMG) significantly disturbed microbiome balance, decreasing bacterial richness, diversity, and uniformity. This suggests that salivary components from the SMG and SLG have considerable effects on oral microbial homeostasis, with SMG playing the major role.

Previous studies reported that alteration to the salivary constituents can result in oral dysbacteriosis. Here, we manipulated constituents of saliva by removing the SMG or SLG. *Lactobacillus*, a major contributor to dental caries (Wen et al., 2022), was significantly more abundant in both removal groups and showed strong acid tolerance. *Lactobacillus* has been shown to survive strong acidity. We speculated that this increase in relative abundance might be related to the low-pH of saliva after SMG or SLG removal.

The SMG mainly consists of seromucous cells that generate neutral secretions. We performed a proteomic assay. The result suggest that carbonic anhydrase VI (CA6), which is related to pH homeostasis (Esberg et al., 2019), is more abundant in the SMG. In a previous study, 13 enzymatically active CA isozymes were identified. CA6 is the only secreted isozyme (Kivela et al., 1999). CAs are zinc enzymes that catalyze the reversible hydration of carbon dioxide, and are important for pH homeostasis in saliva (Supuran, 2008). In the SMG-removal group, the pH imbalance may have occurred due to decreased CA6 protein levels. Thus, the SMG could be a key organ in prevention of oral diseases such as dental caries and periodontitis.

The SLG comprises a large proportion of mucous cells that secrete acidic mucus, as illustrated by alcian blue-periodic acid-Schiff (AB-PAS) staining. In the SLG-removal group, *Lactobacillus* was even more abundant than in the SMG-removal group. We posited that this relative abundance might be the result of several factors, such as saliva flow rate, other unknown proteins, and interactions related to bacterial growth (commensalism or competition).

Six groups of *Streptococcus* (*S. mitis*, *sanguinis*, *anginosus*, *salivarius*, *downei*, and *mutans*) are common in the oral cavity and significantly associated with oral health (Abranches et al., 2018). Among these, *S. mutans* is directly associated with development of dental caries (Moye et al., 2014). In this study, *Streptococcus* was more abundant in the SMG-removal group than that in the sham-operated group. Secreted proteins such as PIP and MUC19 are involved in *Streptococcus* regulation (Culp et al., 2015). Proteomic quantitative analysis found more PIP in SMG and more MUC19 in SLG. PIP and MUC19 showed high affinity for *Streptococcus* and had considerable effects on its colonization (Lee et al., 2002). Both can also attenuate dental infections by assisting *S. mutans* aggregation and clearance, representing an oral defense mechanism in salivary constituents (Lee et al., 2002; Culp et al., 2015). MUC19 controls the initial adherence of bacteria to tooth surfaces, provides bacteria with nutrients, and participates in the innate immune system (Culp et al., 2015). Interestingly, *Streptococcus* did not change drastically in the SLG-removal group. We believe that MUC19 alone cannot lead to dominant *Streptococcus* colonization, as this requires the cooperation of multiple proteins.

*Desulfovibrio* species are gram-negative bacteria that use sulfate compounds as terminal electron acceptors in their respiratory chain (Tchinda et al., 2021). In humans, *Desulfovibrio* species (*D. fairfieldensis*, *D. piger*, and *D. vulgaris*) have been found in the oral cavity and reportedly cause infection (Tee et al., 1996). In periodontitis patients, *D. fairfieldensis* is found in periodontal pockets, where it produces H2S owing to its sulfate-reducing activity. H2S causes direct toxicity to collagen fibers, indirectly disrupting immunological defense (Loubinoux et al., 2002). We found that *Desulfovibrio* abundance increased in the SLG-removal group but decreased in the SMG-removal group. This result suggested that some proteins secreted by the SLG might inhibit growth of *Desulfovibrio* species.

Several antimicrobial proteins could play important roles in oral microbiota homeostasis. In the SMG, KEGG and IPR enrichment suggests that LPO participate in innate immunity. LPO, a heme-containing glycoprotein that catalyzes hydrogen peroxide-dependent oxidation of thiocyanate to hypothiocyanite, has bactericidal effects on periodontal pathogenic bacteria and reduces oral malodor (Welk et al., 2021). Lactoperoxidase exhibits membrane permeabilization activity against gram-positive and gram-negative bacteria (Morita et al., 2017; Manabu et al., 2017). Therefore, LPO also plays a major role in clearing the oral mucosa from various biological threats, preserving homeostasis of the oral cavity. Meanwhile, SCGB2B26, SCGB1B27, SCGB2B27, and OVOS are involved in acquired immunity, which also effects the oral microflora. SCGB2B26, SCGB1B27, and SCGB2B27 were more abundant in the SMG. These secretoglobins play an important role in maintaining microbial homeostasis by binding to surface molecules of pathogenic microorganisms, such as adhesins, preventing them from adhering to the oral mucosa (Gibbins et al., 2015). OVOS, an immunoglobulin, was more abundant in the SLG. Micropia control drugs, which include these antimicrobial proteins, could potentially be administered to the oral cavity when the secretory function of the SMG/SLG is impaired to prevent oral diseases caused by oral microbial dysbiosis.

We performed scRNA-seq analysis to locate cells that secrete antimicrobial proteins. Previous studies reported that the characteristic spectrum of salivary proteins are produced in mucous and serous acinar cells. Recently, the heterogeneity of acinar cells was characterized. Each type of acinar cell synthesizes unique salivary proteins. In our study, cells expressing microbiota homeostasis-regulatory proteins in the SMG were categorized into three serous acinar subpopulations, namely Cluster 0, 2, and 12. Positive expression of *Pip* was observed in all three serous acinar subpopulations, whereas the positive expression of *Ca6* was observed in only Cluster 0 and 2. All cells expressing *Lpo*, *Scgb2b27*, *Scgb2b26*, *Scgb1b27*, *Pip*, and *Ca6* separately were centered on Cluster 2 alone. These results suggest that this common subpopulation might be key for maintaining oral microbiota homeostasis. In the SLG, cells expressing genes encoding microbiota homeostasis-regulatory proteins, namely *A2ml1* and *Muc19*, were categorized into common mucous acinar subpopulation, Cluster 10. Interestingly, *Agr2*, *Pdia5*, *Txndc5*, and *Cgref1* were also highly expressed in this subpopulation. Whether these proteins play a role in maintaining oral microbiota homeostasis is unknown.

Our results also showed that ductal epithelial, mesenchymal, and immune cells express genes that encode microbiota homeostasis-regulatory proteins. Interestingly, these cells are all found in the SMG, which could explain why SMG removal caused more severe oral microbiota imbalance. In the SLG, only the mucinous acinar cells are responsible for encoding microbiota homeostasis-regulatory proteins. However, it is possible that there are more functional factors produced by non-acinar cells in the SLG.

Under extreme circumstances, salivary glands (especially acinar cells) may be severely damaged, for example during radiation treatment for head and neck cancer or Sjögren’s syndrome. This causes dysbiosis of the oral microbiome and severe complications to oral health, decreasing the patient’s quality of life (Saitou et al., 2020). Our results suggest that non-acinar cells, such as ductal epithelial cells, mesenchymal cells, and immune cells, could partially substitute acinar cells in producing microbial regulatory factors during mild gland dysfunction.

In summary, our results reveal the role of the SMG and SLG in the maintenance of oral microbial homeostasis. Our study could form the basis for further research on the prevention of oral diseases caused by oral microbial dysbiosis.

## Data availability statement

The datasets presented in this study can be found in online repositories. The names of the repository/repositories and accession number(s) can be found below: 16s RNA data is located at: https://www.ncbi.nlm.nih.gov/bioproject/PRJNA890588; Or could be accessed by BioProject ID PRJNA890588. Our original single-cell RNA sequencing data can be accessed with GSE216476 (https://www.ncbi.nlm.nih.gov/geo/query/acc.cgi?acc=GSE216476). Additionally, we made the proteomics data available to public at ProteomeXchange (http://www.ebi.ac.uk/pride) with Project Webpage: http://www.ebi.ac.uk/pride/archive/projects/PXD038423; FTP Download: ftp://ftp.pride.ebi.ac.uk/pride/data/archive/2023/01/PXD038423.

## Ethics statement

The animal study was reviewed and approved by Laboratory Animal Welfare (GB/T35892-2018, China).

## Author contributions

LC and TZ contributed to design and critically revised manuscript. YL contributed to data acquisition, analysis, and interpretation, drafted and critically revised the manuscript. JL, YZ, QC, HL, and CL contributed to data acquisition and drafted the manuscript. TG contributed to analysis and drafted the manuscript. HC and WL contributed to analysis and interpretation and critically revised manuscript. All authors gave their final approval and agree to be accountable for all aspects of the work.
