# Supplementary material for: The submandibular and sublingual glands maintain oral microbial homeostasis through multiple antimicrobial proteins
Source: Front Cell Infect Microbiol. 2023 Jan 10;12:1057327. doi: 10.3389/fcimb.2022.1057327 (PMC9872150; doi:10.3389/fcimb.2022.1057327)
Supplement: Supplementary file 1 [file DataSheet_1.docx]

**Appendix. Figure 1 Rarefaction curves and the total OTUs.**

**
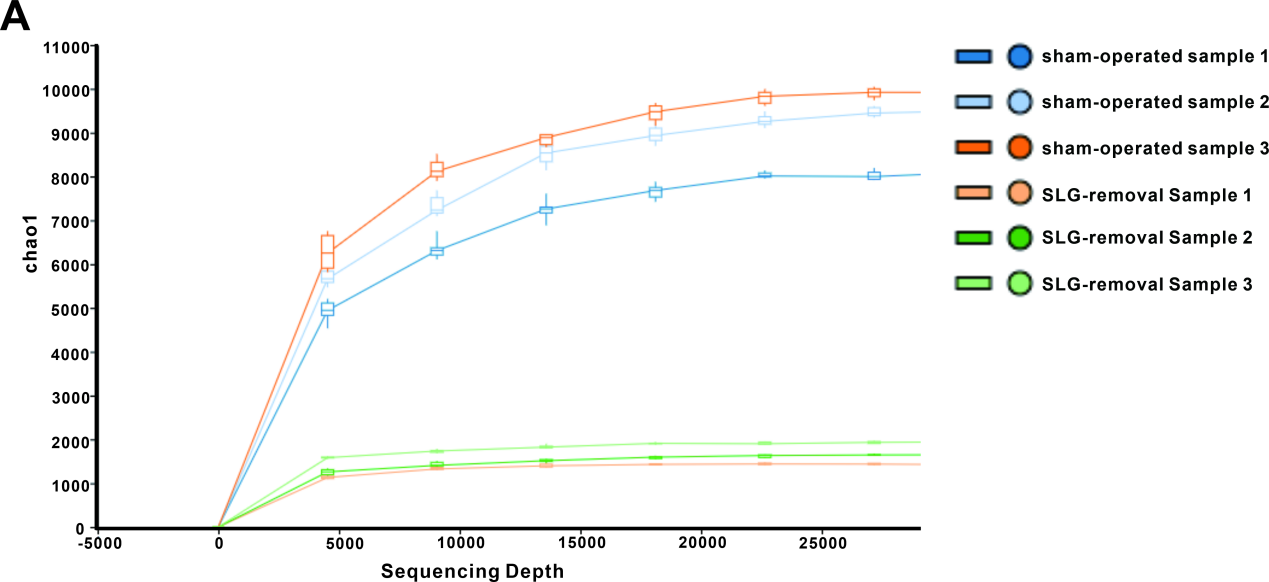
**

**
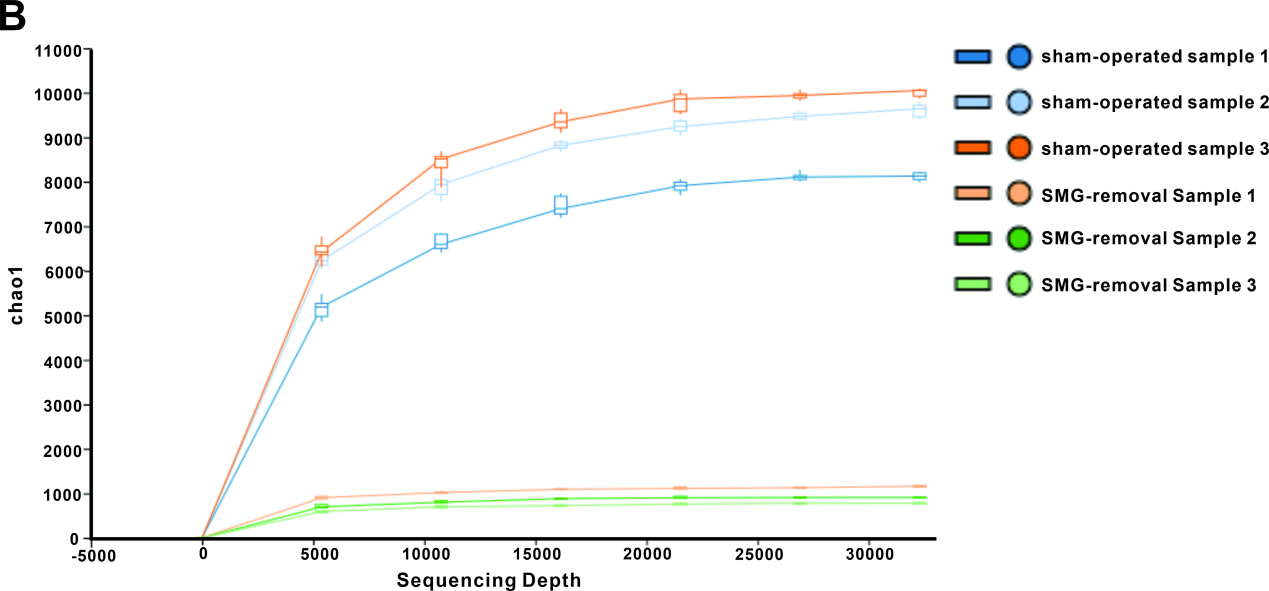
**

**C.**

| **Group** | **Sample** | **OTUs** |
| --- | --- | --- |
| **Sham-operated** | **Sample1** | **6463** |
|  | **Sample2** | **7390** |
|  | **Sample3** | **7954** |
| **SLG-removal** | **Sample1** | **1403** |
|  | **Sample2** | **1560** |
|  | **Sample3** | **1883** |
| **SMG-removal** | **Sample1** | **1082** |
|  | **Sample2** | **856** |
|  | **Sample3** | **732** |

**Appendix. Figure 1**: **(A)**Rarefaction curves of samples in sham-operated and SLG-removal group. **(B)** Rarefaction curves of samples in sham-operated and SMG-removal group. **(C)** The total OTUs in samples from sham vs SMG and SLG.

**Appendix. Table 1 qPCR primers used in this study.**

| Gene | Sequence |
| --- | --- |
| Col1a2 | (F) 5’- CTATTGGAAGCCGAGGTCC - 3’  (R) 5’- CAGCACCCCTCTCTCCTG - 3’ |
| Scgb2b27 | (F) 5’- CTACTGCAGCGGAAAGGGT - 3’  (R) 5’- TCTGGAAGGCATGATGAGGG - 3’ |
| Pip | (F) 5’- CTATGTCTGCAGCTGGGGAT - 3’  (R) 5’-TTCCTGTGCTGTTGATGGAA - 3’ |
| Cd44 | (F) 5’- CTTGGGGACTTTGCCTCTTG - 3’  (R) 5’- GGAGATACTGTAGCGGCCAT - 3’ |
| Ctsd | (F) 5’- ACTACGGAGGTGAACGACTAC - 3’  (R) 5’- TCCTCCACAGCTCCATTCAG - 3’ |
| Scgb1b27 | (F) 5’- CTACTGCAGCGGAAAGGGT - 3’  (R) 5’- TCTGGAAGGCATGATGAGGG - 3’ |
| Lpo | (F) 5’- CTTCCCTGACCTTGCTCCAGAC - 3’  (R) 5’- GACCCAGACCTTGACCTCTTCC - 3’ |
| A2ml1 | (F) 5’- TTTGCAACTGGAACTCGACG - 3’  (R) 5’- AACGATGGCAGCTTCTAGGT - 3 |
| Muc19 | (F) 5’- AATCAACTTGCCATGTCTACGG - 3’  (R) 5’- CTTGGCCACAGTAATCCTCAAG - 3’ |
| Scgb2b26 | (F) 5’- GTAGCTGAAAGGGTGGCCTT - 3’  (R) 5’- TCTTGCATTCTGGGCTTAAGT - 3’ |
| Pigr | (F) 5’- CTACAAGTGTGGCCTGGGTA - 3’  (R) 5’- TCCTTTGTGTAGACGTGGGT - 3’ |
| Mucl2 | (F) 5’- CTGCTGATTCTGCTGGTGAA - 3’  (R) 5’- GGACTCGTCTTCCGTTTCAG - 3’ |
| Ca6 | (F) 5’- GCCCTCCATGTACCTTGAAA - 3’  (R) 5’- GACGGCTAACACAGCTAGGC - 3’ |
| β-Actin | (F) 5’- TCTTTGCAGCTCCTTCGTTG - 3’  (R) 5’- CGATGGAGGGGAATACAGCC - 3’ |

**Appendix. Table2 The** **results of** **the** **blood test in mouse model.**

|  | sham-  operated 1 | sham-  operated 2 | sham-  operated 3 | removal SMG1 | removal SMG2 | removal SMG3 | removal SLG1 | removal SLG2 | removal SLG3 |
| --- | --- | --- | --- | --- | --- | --- | --- | --- | --- |
| WBC | 5.97 | 3.08 | 4.4 | 3.92 | 5.46 | 13.86 | 2.59 | 7.03 | 6.18 |
| RBC | 9.8 | 9.4 | 10.49 | 10.65 | 9.76 | 10.76 | 9.79 | 10.61 | 9.98 |
| Hb | 140 | 133 | 139 | 142 | 129 | 147 | 133 | 144 | 131 |
| HCT | 48 | 47.6 | 48.9 | 50.7 | 47.3 | 52 | 48 | 49.2 | 44.7 |
| PLT | 1718 | 1675 | 2506 | 1952 | 979 | 1081 | 1603 | 1042 | 1157 |
| MPV | 7 | 7.3 | 6.9 | 7 | 7.2 | 7.5 | 7.2 | 6.8 | 7.3 |
| PCT | 1.21 | 1.22 | 1.73 | 1.37 | 0.7 | 0.82 | 1.15 | 0.71 | 0.84 |
| MCV | 49 | 50.6 | 46.6 | 47.6 | 48.5 | 48.3 | 49 | 46.4 | 44.8 |
| MCH | 14.3 | 14.1 | 13.3 | 13.3 | 13.2 | 13.7 | 13.6 | 13.6 | 13.1 |
| MCHC | 292 | 279 | 284 | 280 | 273 | 283 | 277 | 293 | 293 |
| NEU(%) | 23.4 | 34.1 | 39.8 | 36.5 | 24.7 | 11.94 | 42.1 | 15.34 | 26.5 |
| LYM(%) | 69.3 | 53.2 | 56.4 | 59.7 | 72.2 | 85.54 | 47.9 | 77.84 | 60.7 |
| MON(%) | 5 | 10.1 | 3.6 | 1.5↓ | 2.4↓ | 1.44↓ | 7.3 | 3.84 | 11.5 |
| EOS(%) | 2.3 | 2.6 | 0.2 | 2.3 | 0.7 | 1.24 | 2.3 | 3.04 | 1.1 |
| BAS(%) | 0 | 0 | 0 | 0 | 0 | 40 | 400↑ | 140↑ | 200↑ |
| NEU | 1.39 | 1.05 | 1.75 | 1.43 | 1.35 | 1.66 | 1.09 | 1.07 | 1.64 |
| LYM | 4.14 | 1.64 | 2.48 | 2.34 | 3.94 | 11.85 | 1.24 | 5.47 | 3.75 |
| MON | 0.3 | 0.31 | 0.16 | 0.06↓ | 0.13↓ | 0.19↓ | 0.19 | 0.27 | 0.71 |
| EOS | 0.14 | 0.08 | 0.01 | 0.09 | 0.04 | 0.16 | 0.06 | 0.21 | 0.07 |
| BAS | 0 | 0 | 0 | 0 | 0 | 0.4 | 1↑ | 1.4↑ | 1↑ |
